# Population-based nasopharyngeal carcinoma survival in southern China

**DOI:** 10.1038/s41416-025-03232-w

**Published:** 2025-10-29

**Authors:** Yun Du, Ruimei Feng, Ellen T. Chang, Li Yin, Paul Dickman, Tingting Huang, Yancheng Li, Xiang Zhou, Yi Huang, Canqiong Su, Xue Xiao, Weihua Jia, Yuming Zheng, Hans-Olov Adami, Yixin Zeng, Yonglin Cai, Zhe Zhang, Miao Xu, Weimin Ye

**Affiliations:** 1 Department of Medical Epidemiology and Biostatistics, Karolinska Institutet, Stockholm, Sweden; 2 Department of Epidemiology and Health Statistics and Key Laboratory of Ministry of Education for Gastrointestinal Cancer, Fujian Medical University, Fuzhou, China; 3 Department of Epidemiology and Biostatistics, University of California San Francisco, San Francisco, CA USA; 4 Department of Radiation Oncology, The First Affiliated Hospital of Guangxi Medical University, Nanning, China; 5 Key Laboratory of High-Incidence-Tumor Prevention & Treatment (Guangxi Medical University), Ministry of Education, Nanning, China; 6 Guangxi Health Commission Key Laboratory of Molecular Epidemiology of Nasopharyngeal Carcinoma, Wuzhou Red Cross Hospital, Wuzhou, China; 7 State Key Laboratory of Oncology in South China, Collaborative Innovation Center for Cancer Medicine, Guangdong Key Laboratory of Nasopharyngeal Carcinoma Diagnosis and Therapy, Sun Yat-sen University Cancer Center, Guangzhou, China; 8 Department of Otolaryngology-Head & Neck Surgery, First Affiliated Hospital of Guangxi Medical University, Nanning, China; 9 Clinical Effectiveness Group, Institute of Health and Society, University of Oslo, Oslo, Norway; 10 Present Address: Department of Oncology, Southwest Hospital, Third Military Medical University, Chongqing, China; 11 Present Address: Department of Epidemiology, School of Public Health, Shanxi Medical University, Jinzhong, 030606 China

**Keywords:** Epidemiology, Head and neck cancer

## Abstract

**Background:**

Few population-based studies on nasopharyngeal carcinoma (NPC) prognosis exist. Such real-world data with complete follow-up are needed to enable accurate estimation of survival probabilities and monitor progress in early detection and treatment.

**Methods:**

In a population-based study of 2529 patients with incident NPC in southern China diagnosed in 2010–2013, we developed a passive-active-passive follow-up strategy involving data linkage and direct contact to track patients’ vital status, cause of death, and migration through December 31, 2018. We calculated 5-year survival probabilities by the Kaplan-Meier method, and estimated avoidable deaths comparing hypothetical scenarios and real-world observations.

**Results:**

Early-stage (I–II) patients accounted for 11·5%, and 21·1% were treated in medical-university-affiliated or province-level hospitals. With a mean follow-up time of 5·5 years after diagnosis (98.3% complete), 5-year overall and NPC-specific survival probabilities were 70·1% and 74·5%. The 5-year overall survival probabilities for stage I, II, III, IVa, IVb, and IVc were 91·3%, 87·8%, 79·8%, 63·9%, 57·7%, and 34·4%, respectively. Survival of non-metastatic (I-IVb) population-based patients diagnosed in 2010–2013 was comparable to that of a hospital-based southern Chinese NPC cohort diagnosed in 1997–2007. Based on observed data, if all patients were diagnosed at stages I–II, then total avoidable deaths within 5 years after diagnosis would be 156 per 1000 patients.

**Conclusions:**

Real-world population-based NPC survival lags behind that of large hospital-based cohorts, but earlier diagnosis has the potential to substantially reduce mortality from NPC. Our results highlight the importance of improving the accessibility of health resources and promoting early detection of NPC.

## Introduction

The incidence of nasopharyngeal carcinoma (NPC) in southern China is the highest worldwide [1, 2]. Radiotherapy, with or without chemotherapy, is the primary treatment modality for NPC. Advances in radiotherapy techniques over time, along with improved efficacy of chemotherapy and immunotherapy, have improved NPC survival rates. For example, in recent hospital-based studies, the 5-year overall survival rate for all stages of NPC combined was over 80% [3, 4]. However, hospital-based survival probabilities reflect the treatment quality provided in particular hospitals, and typically do not reveal the effectiveness of cancer control in an entire geographic region. In addition, for NPC follow-up in hospitals, data completeness relies on patients’ compliance with monitoring recommendations [4]. Thus, loss to follow-up is typically high, resulting in biased estimation of survival probabilities [5].

Few studies have reported population-based NPC survival rates. One study in southern China between 1976 and 2005 investigated NPC survival at a population level [6], but utilized only passive follow-up through a regional cancer registry, which is prone to incomplete follow-up due to lagging death registration [7]. Another population-based study in Taiwan between 2002 and 2010 did not report the rate of loss to follow-up [8]. Neither of these studies reported stage-specific survival for cases classified according to a unified staging system, nor did they report detailed treatment types. Given the scarcity of population-based data, it is crucial to gain a better understanding of the current state of NPC survival to better inform disease management strategies.

To address this knowledge gap, we conducted a population-based study of NPC survival in southern China. Our study included the development of a follow-up strategy to achieve high follow-up completeness, to better quantify the landscape of NPC survival in southern China, as well as compare observed survival rates with those in other NPC-endemic populations, and calculate the proportion of deaths potentially avoidable through earlier detection or better treatment access.

## Methods

### Study population

This population-based survival study in NPC-endemic regions of southern China was based on the patient cohort from our project, NPCGEE, described in detail previously [9]. Briefly, the study base consisted of 13 cities/counties with approximately 8 million residents from three regions: Wuzhou and Guiping/Pingnan in Guangxi Autonomous Region, and Zhaoqing in Guangdong Province. From March 2010 to December 2013, we enrolled incident NPC cases aged between 20 and 74 years, without a history of cancer or congenital or acquired immune deficiency, and able to finish the study interview. We identified 3047 newly diagnosed cases, of whom 2553 (83.8%) were enrolled. After excluding subjects not confirmed as incident NPC based on subsequent review of medical records, we retained 2529 cases in the final analysis (Fig. S1).

### Covariates

Covariate information was obtained by face-to-face interview and medical record review. The interview was based on a structured questionnaire at diagnosis. Tobacco use was categorized as never, former, or current smoker [10]. We classified education according to the Chinese education system [11]: illiterate/primary school, middle school, high school, or vocational or technical college/university and above. Occupation was classified as farmer, blue-collar, white-collar, unemployed, or unknown/other.

Medical records were reviewed by trained medical personnel. Clinical stage was restaged according to the 7th Edition of the American Joint Committee on Cancer [12]. Histopathological classification was categorized as nonkeratinizing carcinoma or other (keratinizing squamous cell carcinoma and basaloid squamous cell carcinoma), based on 2005 WHO tumor classification [13]. Karnofsky Performance Scale (KPS) was grouped into two levels (<90 and ≥90). Body mass index (BMI) was categorized based on WHO guidelines for Asians [14]: underweight (<18.5 kg/m^2^), normal weight (18.5–22.9 kg/m^2^), overweight (23.0–27.4 kg/m^2^), or obese (≥27.5 kg/m^2^). We divided treatment hospitals into medical-university-affiliated/province-level and prefecture-level groups. We categorized treatment patterns according to clinical routine: concurrent chemoradiotherapy (CCRT), CCRT with adjuvant chemotherapy (ACT) and/or induction chemotherapy (ICT), only radiotherapy, only chemotherapy, neither radiotherapy nor chemotherapy, or radiotherapy and ACT/ICT. Radiotherapy technique was classified as two-dimensional radiotherapy (2DRT), three-dimensional radiotherapy (3DRT), or intensity-­modulated radiotherapy (IMRT).

### Follow-up

NPC patients were followed for vital status, cause of death, and migration out of the study area. We used a passive-active-passive circle follow-up strategy, adopted based on the practical considerations given the study’s geographic setting (Figs. S2 and S3). The first step in this strategy entailed passive follow-up through linkage of the cohort to population databases, including regional cancer registries, the cause-of-death registry, the medical insurance system, and the total population registry, as well as to hospital medical records. For cases identified from this initial step as being lost to follow-up or deceased without a known cause of death, we conducted a second, active follow-up step by attempting to contact the patients or their relatives through phone calls or in-person home visits by village doctors. Finally, we performed passive and active follow-ups repeatedly to ascertain outcomes for the remaining cases.

Follow-up time was calculated from the date of diagnosis to 31 December 2018, migration out of the study area, or death, whichever occurred first. For two deceased cases without a known date of death, we assigned the median survival time of deceased cases in the corresponding geographic area, stage, and age. For seven patients lost to follow-up immediately after the date of diagnosis (i.e., follow-up = 0), we set the follow-up time to a half-day.

### Statistical analyses

Five-year survival probability was calculated using the Kaplan-Meier method, and differences between categories were tested using the log-rank test. To identify comparable survival statistics previously reported in NPC-endemic areas, we searched the PubMed database and Web of Science, as well as bibliographies, using the search strategy presented in Methods S1.

To calculate the theoretical number of avoidable deaths due to earlier-stage diagnosis or treatment in university-affiliated or province-level hospitals, we used flexible parametric models with the R package *rstpm2* [15], as described in Methods S2.

For 165 deceased cases with unknown cause of death, we performed multiple imputation (Methods S2 and S3) and obtained the NPC-specific survival probabilities and avoidable NPC-specific deaths.

### Supplementary analysis

To assess the robustness of results from multiple imputation, we did extreme single-imputations, for example, assuming that the 165 decedents with an unknown cause of death were deceased from either NPC or any other cause.

To quantify competing risks, we calculated Aalen-Johansen estimates (cumulative incidence functions) of NPC cause-specific survival, treating the 165 decedents with an unknown cause of death as deceased due to either NPC or other causes.

To present factors associated with NPC survival, we used Cox proportional hazards regression to calculate crude and adjusted all-cause and NPC-specific hazard ratios (HRs) in relation to demographic and clinical characteristics.

All statistical analyses were performed using R (version 4.0.3), with two-sided tests and a significance level of *α* = 0.05.

## Results

### Patient characteristics

The study cohort comprised 2529 patients with an incident NPC, including 1300 in Zhaoqing, 546 in Guiping/Pingnan, and 683 in Wuzhou (Table 1). Early-stage cases (I and II) accounted for only 11.5% of the cohort. One-fifth (21.1%) were treated in university-affiliated/province-level hospitals, whereas 72.1% received treatment in prefecture-level hospitals; the remainder had unknown treatment location. The most common radiotherapy technique was 2DRT (50.8%). The mean follow-up time was 5.5 years. The proportion of patients lost to follow-up was 1.7%. Overall, 74.4% of patients who were still alive at the end of follow-up were captured by the active follow-up method, whereas 72.3% of deceased patients were captured by the passive follow-up method. Causes of death were ascertained by active follow-up for 73.4% of the deceased; in Zhaoqing, 64.7% of causes of death were obtained by passive follow-up (Fig. S3B).

**Table 1:** Characteristics of primary NPC cases during 2010-2013 in southern China by residential area.

Characteristics	Zhaoqing (*N* = 1300)	Guiping/Pingnan (*N* = 546)	Wuzhou (*N* = 683)	Total (*N* = 2529)
Age at cancer diagnosis, years				
Mean (SD)	47.9 (10.7)	49.7 (10.9)	48.8 (10.4)	48.5 (10.7)
Sex, *n* (%)				
Female	349 (26.8%)	142 (26.0%)	182 (26.6%)	673 (26.6%)
Male	951 (73.2%)	404 (74.0%)	501 (73.4%)	1856 (73.4%)
Marital status at diagnosis, n (%)				
Married	1221 (93.9%)	514 (94.1%)	649 (95.0%)	2384 (94.3%)
Not married	79 (6.1%)	32 (5.9%)	34 (5.0%)	145 (5.7%)
Educational attainment, *n* (%)				
Illiterate/Primary school	478 (36.8%)	241 (44.1%)	286 (41.9%)	1005 (39.7%)
Middle school	558 (42.9%)	210 (38.5%)	243 (35.6%)	1011 (40.0%)
High school	204 (15.7%)	76 (13.9%)	127 (18.6%)	407 (16.1%)
Vocational or technical college/University and above	60 (4.6%)	19 (3.5%)	27 (4.0%)	106 (4.2%)
Occupation at diagnosis, *n* (%)				
Farmer	388 (29.8%)	246 (45.1%)	218 (31.9%)	852 (33.7%)
Blue collar	570 (43.8%)	179 (32.8%)	273 (40.0%)	1022 (40.4%)
White collar	195 (15.0%)	52 (9.5%)	103 (15.1%)	350 (13.8%)
Unemployed	43 (3.3%)	16 (2.9%)	18 (2.6%)	77 (3.0%)
Unknown/other	104 (8.0%)	53 (9.7%)	71 (10.4%)	228 (9.0%)
Smoking history, *n* (%)				
Never	545 (41.9%)	254 (46.5%)	317 (46.4%)	1116 (44.1%)
Former	111 (8.5%)	30 (5.5%)	38 (5.6%)	179 (7.1%)
Current	638 (49.1%)	262 (48.0%)	328 (48.0%)	1228 (48.6%)
Missing	6 (0.5%)	0 (0%)	0 (0%)	6 (0.2%)
Treatment hospital, *n* (%)				
Medical university-affiliated/province-level	183 (14.1%)	315 (57.7%)	35 (5.1%)	533 (21.1%)
Prefecture-level	1026 (78.9%)	168 (30.8%)	629 (92.1%)	1823 (72.1%)
Missing	91 (7.0%)	63 (11.5%)	19 (2.8%)	173 (6.8%)
BMI before treatment, *n* (%)				
Normal weight	616 (47.4%)	233 (42.7%)	352 (51.5%)	1201 (47.5%)
Underweight	175 (13.5%)	45 (8.2%)	98 (14.3%)	318 (12.6%)
Overweight	335 (25.8%)	138 (25.3%)	181 (26.5%)	654 (25.9%)
Obese	63 (4.8%)	27 (4.9%)	30 (4.4%)	120 (4.7%)
Missing	111 (8.5%)	103 (18.9%)	22 (3.2%)	236 (9.3%)
KPS before treatment, *n* (%)				
<90	170 (13.1%)	46 (8.4%)	96 (14.1%)	312 (12.3%)
≥90	1029 (79.2%)	392 (71.8%)	560 (82.0%)	1981 (78.3%)
Missing	101 (7.8%)	108 (19.8%)	27 (4.0%)	236 (9.3%)
Histological type, *n* (%)				
Others	39 (3.0%)	30 (5.5%)	22 (3.2%)	91 (3.6%)
Non-keratinizing carcinoma	1171 (90.1%)	460 (84.2%)	635 (93.0%)	2266 (89.6%)
Missing	90 (6.9%)	56 (10.3%)	26 (3.8%)	172 (6.8%)
Clinical stage at diagnosis, *n* (%)				
I	19 (1.5%)	15 (2.7%)	13 (1.9%)	47 (1.9%)
II	154 (11.8%)	45 (8.2%)	43 (6.3%)	242 (9.6%)
III	530 (40.8%)	205 (37.5%)	274 (40.1%)	1009 (39.9%)
IVa	288 (22.2%)	114 (20.9%)	183 (26.8%)	585 (23.1%)
IVb	154 (11.8%)	70 (12.8%)	115 (16.8%)	339 (13.4%)
IVc	62 (4.8%)	33 (6.0%)	34 (5.0%)	129 (5.1%)
Missing	93 (7.2%)	64 (11.7%)	21 (3.1%)	178 (7.0%)
Treatment pattern, *n* (%)				
CCRT	484 (37.2%)	204 (37.4%)	351 (51.4%)	1039 (41.1%)
CCRT + ICT/ACT	561 (43.2%)	181 (33.2%)	215 (31.5%)	957 (37.8%)
RT only	96 (7.4%)	47 (8.6%)	68 (10.0%)	211 (8.3%)
Chemo only	45 (3.5%)	13 (2.4%)	19 (2.8%)	77 (3.0%)
Neither RT nor CT	26 (2.0%)	15 (2.7%)	11 (1.6%)	52 (2.1%)
RT + ICT/ACT	0 (0%)	2 (0.4%)	0 (0%)	2 (0.1%)
Missing	88 (6.8%)	84 (15.4%)	19 (2.8%)	191 (7.6%)
RT technique, *n* (%)				
2DRT	729 (56.1%)	159 (29.1%)	396 (58.0%)	1284 (50.8%)
3DRT	107 (8.2%)	32 (5.9%)	1 (0.1%)	140 (5.5%)
IMRT	300 (23.1%)	248 (45.4%)	236 (34.6%)	784 (31.0%)
No RT	71 (5.5%)	28 (5.1%)	30 (4.4%)	129 (5.1%)
Unknown technique	5 (0.4%)	18 (3.3%)	1 (0.1%)	24 (0.9%)
Missing	88 (6.8%)	61 (11.2%)	19 (2.8%)	168 (6.6%)
Follow-up years				
Mean (SD)	5.67 (2.32)	5.19 (2.20)	5.42 (2.23)	5.50 (2.27)
Survival status at end of follow-up/causes of death, *n* (%)				
Alive	786 (60.5%)	309 (56.6%)	371 (54.3%)	1466 (58.0%)
Death from NPC	267 (20.5%)	176 (32.2%)	258 (37.8%)	701 (27.7%)
Death from other causes	67 (5.2%)	45 (8.2%)	41 (6.0%)	153 (6.0%)
Death from unknown causes	147 (11.3%)	7 (1.3%)	11 (1.6%)	165 (6.5%)
Loss to follow-up	33 (2.5%)	9 (1.6%)	2 (0.3%)	44 (1.7%)

*NPC* nasopharyngeal carcinoma, *EBV* Epstein-Barr virus, *SD* standard deviation, *BMI* body mass index, *KPS* Karnofsky performance scale, *CCRT* concurrent chemoradiotherapy, *ICT* induction chemotherapy, *ACT* adjuvant chemotherapy, *RT* radiotherapy, Chemo chemotherapy, *2DRT* two-dimensional radiotherapy, *3DRT* three-dimensional radiotherapy, *IMRT* intensity-modulated radiotherapy.

### Survival probabilities

**Fig. 1:**
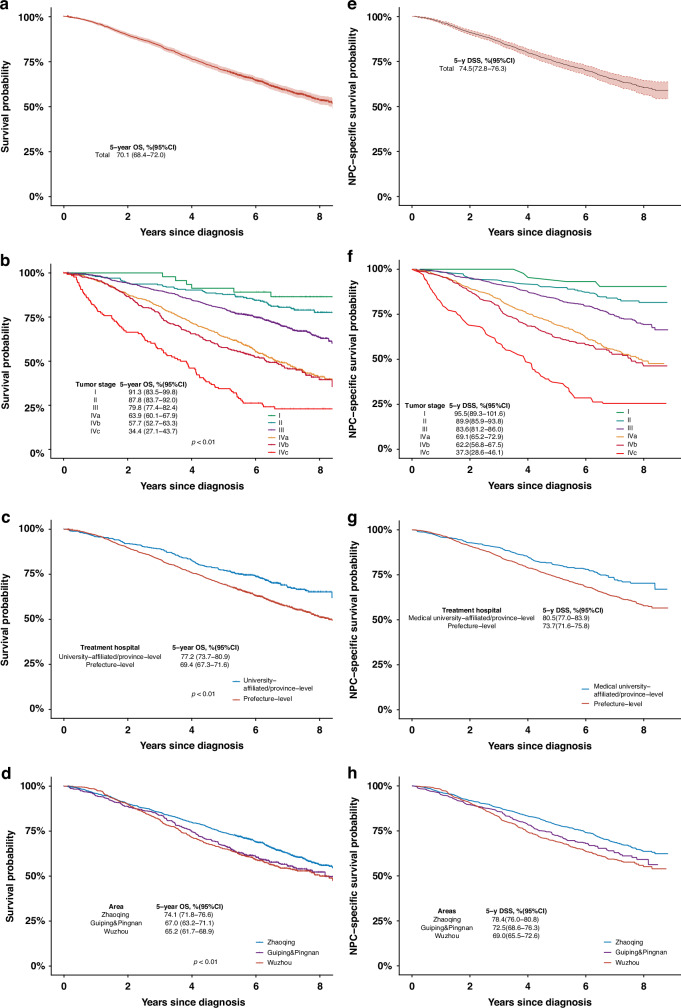
Kaplan-Meier estimates of overall and pooled NPC-specific survival curves of NPC. Kaplan-Meier estimates of overall (**a**–**d**) and pooled NPC-specific (**e**–**h**) survival curves of NPC cases (165 deceased case with unknown cause were imputed by multiple imputation). **a** overall survival for all cases; **b** overall survival by clinical stage at diagnosis; **c** overall survival by types of treatment hospital; **d** overall survival by areas. E NPC-specific survival for all cases; **f** NPC-specific survival by clinical stage at diagnosis; **g** NPC-specific survival by types of treatment hospital; **h** NPC-specific survival by areas. NPC nasopharyngeal carcinoma, DDS disease/NPC-specific survival.

**Fig. 2:**
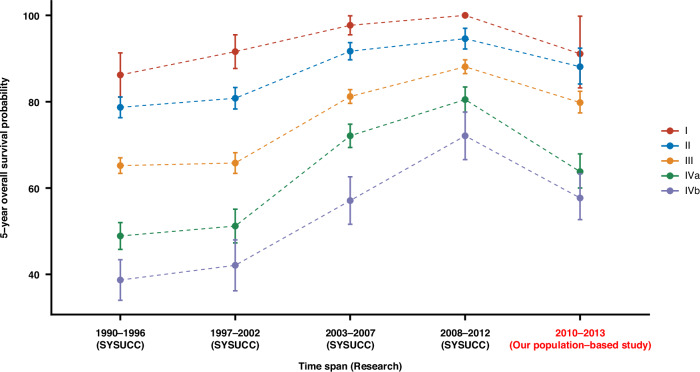
Comparison of 5-year overall survival by stage at diagnosis across calendar periods within a large hospital-based NPC cohort in southern China (SYSUCC).

The 5-year overall and NPC-specific survival probabilities were 70.1% and 74.5% (Fig. 1a, e). Five-year overall and NPC-specific survival by tumor stage at diagnosis were 91.3% and 95.5%, respectively, for stage I, 87.8% and 89.9% for stage II, 79.8% and 83.6% for stage III, 63.9% and 69.1% for stage IVa, 57.7% and 62.2% for stage IVb, and 34.4% and 37.3% for stage IVc (Fig. 2b, f). For patients treated at medical-university-affiliated/province-level hospitals, 5-year overall and NPC-specific survival were 77.2% and 80.5%, respectively, while for those treated at prefecture-level hospitals, the corresponding figures were 69.4% and 73.7% (Fig. 2c, g). Five-year overall and NPC-specific survival probabilities were 74.1% and 78.4% in Zhaoqing, 67.0% and 72.5% in Guiping/Pingnan, and 65.2% and 69.0% in Wuzhou (Fig. 1d, h).

### Comparison with NPC survival in other NPC-endemic areas

We identified 17 studies that reported 5-year overall survival for NPC patients in NPC-endemic regions (Table S1). The most directly comparable study was conducted in a hospital-based NPC cohort diagnosed between 1990 and 2012 at Sun Yat-Sen University Cancer Center, a university-affiliated hospital in Guangdong Province (the same province as Zhaoqing, one of our study regions), which included the largest NPC database reported in China, with 20,305 cases [16]. Comparing 5-year survival between our population-based cohort and the above-mentioned hospital-based cohort revealed an ~10-year lag in the survival of population-based NPC patients (Fig. 2).

### Avoidable deaths

**Fig. 3:**
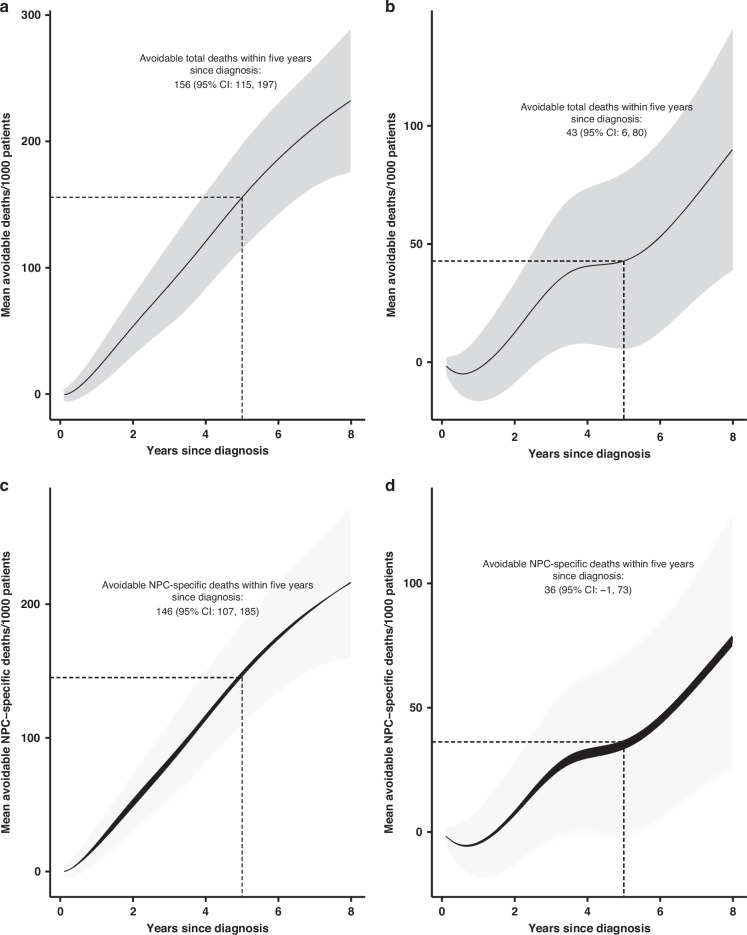
Mean avoidable total deaths and NPC-specific deaths over years since diagnosis, comparing hypothetical with observed scenarios. Mean avoidable total deaths (**a**, **b**) and NPC-specific (**c**, **d**) deaths per 1000 NPC cases by years since diagnosis, comparing hypothetical with observed scenarios. **a** Mean avoidable total deaths between hypothetical scenario if all patients were diagnosed at early stages (I–II) vs. the real-world scenario as observed. **b** Mean avoidable total deaths between hypothetical scenario if all patients were treated in medical university-affiliated or province-level hospitals vs. the real-world scenario as observed. **c** Mean avoidable NPC-specific deaths between hypothetical scenario if all patients were diagnosed at early stages (I–II) vs. the real-world scenario as observed. **d** Mean avoidable NPC-specific deaths between hypothetical scenarios if all patients were treated in medical university-affiliated or province-level hospitals vs. the real-world scenario as observed.

If all NPC cases in our real-world cohort were instead diagnosed at early stages (I–II) as a hypothetical scenario, then the standardized avoidable total deaths and NPC-specific deaths within five years of diagnosis would be 156 (95% CI: 115, 197) and 146 (95%CI: 107, 185) per 1000 patients (Fig. 3a, c). If all patients were hypothetically treated in medical-university-affiliated or province-level hospitals, then the standardized avoidable total deaths and NPC-specific deaths within 5 years after diagnosis would be 43 (95% CI: 6, 80) and 36 (95%CI: −1, 73) per 1000 patients (Fig. 3b, d).

### Supplementary analysis

The 5-year NPC-specifc survival probabilities were 78.4% and 73.8% when all 165 deceased cases with unknown cause of death were classified as dying from non-NPC or NPC, respectively (Fig. S5).

Cumulative 5-year NPC-specific mortality was 0.26 (95% CI: 0.24, 0.28) when the 165 decedents with unknown cause of death were assumed to have died from NPC (Fig. S6As), and 0.21 (95% CI: 0.19, 0.23) when they were assumed to have died from other causes (Fig. S6B).

NPC prognostic factors are shown in Table S2. Cancer stage was the most prominent (i.e., IVc versus I, all-cause HR:11.87, 95% CI: 5.18, 27.21). Being treated with IMRT vs. 2DRT was associated with 31% lower all-cause mortality. Cases treated in prefecture-level hospitals had 42% higher all-cause mortality than cases treated in medical-university-affiliated/province-level hospitals.

## Discussion

We achieved a high follow-up rate of 98.3% in this population-based study with a passive-active-passive circle strategy that combined data linkage with direct outreach to ascertain vital status. In this population-based cohort with mostly late-stage NPC patients treated in prefecture-level hospitals in 2010–2013, 5-year overall survival was 70.1%, comparable to that about 10 years earlier in a large cancer center also based in southern China. We estimated that earlier stage at diagnosis would prevent 156 total deaths per 1000 patients within 5 years after diagnosis, while treatment at medical-university-affiliated or province-level hospitals would save 43 lives per 1000 patients.

Few prior studies have estimated NPC survival in a population-based setting. One such study, conducted in a small city, Sihui in Zhaoqing (part of our study base), included 1761 NPC cases registered from 1976 to 2005 [6]. In this cohort, 61.1% of whom were treated at Sun Yat-sen University Cancer Center and 37% of whom were early-stage (I–II), and the 5-year overall survival probability was 50.5%. In the most recent time period of this study (2000–2005), 5-year survival was 69.8%, comparable to our findings, as was stage-specific 5-year survival (100% based on 7 cases, 87.3%, 66.5%, and 53.5% for stages I, II, III, and IV, respectively); however, the staging systems were not defined and varied over the course of the study period. Another population-based study, including 13,407 NPC cases diagnosed between 2002 and 2010 in Taiwan, reported 5-year overall survival of 65.2%, but lacked information on tumor stage at diagnosis [8].

In the last decade of the 20th century and the beginning of the 21st century, linear accelerator technology made 3DRT and IMRT feasible in China, with obvious therapeutic benefits for patients with NPC; in our cohort 36.5% of the patients received such treatment. The lack of detailed information on radiotherapy technique in prior population-based studies [6, 8] precludes direct comparison with our results. We infer that the observed improvement in survival over time in these studies was most likely due in part to the development of radiation treatment facilities, especially around the beginning of the 21st century. Likewise, within our study population, survival in Zhaoqing was significantly better than in the other two regions, probably due to its better economy and greater access to advanced treatment [17].

Given the importance of modern radiotherapy modalities for NPC, it is unsurprising that treatment hospital was a strong prognostic factor. We estimated that if all patients had access to medical-university-affiliated or province-level hospitals, then 43 deaths would be avoided for every 1000 patients. Radiotherapy needs a team of experts composed of clinical physicians, radiologists, medical radiation physicists, and biologists, as well as advanced radiotherapy facilities and buildings with strict structural requirements to isolate the beam source from the environment. Thus, resources for radiotherapy, especially advanced radiotherapy techniques remain uncommon, in remote prefecture areas, where more than 72% of our population-based patients received treatment.

Another explanation for observed differences in overall survival for NPC is the implementation of screening programs for early diagnosis. We estimated that effective early detection screening would prevent approximately 15% of deaths if all cases were instead diagnosed at stages I–II. Due to the high prevalence of NPC in southern China, as well as strong evidence supporting early detection of NPC using EBV-based biomarkers [18], population-based NPC screening programs were launched in the past 50 years across southern China, especially in Guangdong Province [19–21]. Early results from a randomized controlled screening trial indicate that this strategy increases early detection and reduces NPC mortality [22]. Our finding that Zhaoqing had the highest proportion of early-stage cases may be due to the presence of an NPC screening program in this area [20, 23]. Given the relatively low response rate to NPC screening programs (around 20%) [20] and the strong evidence supporting the benefit of screening [18], more resources are needed to implement NPC screening programs and increase participation rates within endemic regions.

Most previously reported survival figures were hospital-based, usually at medical-university-affiliated or province-level hospitals, with higher survival probabilities than ours in contemporaneous time periods [16, 24, 25]. In general, based on advanced hospitals, the 5-year survival of locally advanced cases diagnosed after 2000 ranged from 76.0% to 86.0% [24–26]. In our population-based setting, only 31.0% of patients were treated by IMRT, whereas 55.0% of NPC patients at Sun Yat-sen University Cancer Center received IMRT treatment during a similar time period (2008–2012) [16]. Moreover, only 21.1% of our population-based cases received treatment at medical-university-affiliated or province-level hospitals, where most prior NPC survival studies were conducted.

To our knowledge, this is the only population-based NPC survival study in an NPC-endemic area with a high enrollment rate (83.8%), nearly complete follow-up (98.3%), and information on unified clinical stage and detailed radiotherapy modality, as well as demographic and environmental risk factors. Thus, our study provides valid, representative, and generalizable information on NPC survival in southern China, which bears the greatest public health burden of NPC worldwide.

Limitations of our study include the lack of information on adverse radiotherapy effects and disease recurrence, precluding an evaluation of quality of life or other non-fatal outcomes among NPC cases. In addition, about 16% of decedents had a missing or unknown cause of death, affecting our analysis of NPC cause-specific survival. Non-random missingness could have modestly biased our results. We performed multiple imputation to estimate the NPC-specific survival and extreme imputation to confirm its robustness. Finally, causes of death may be misclassified, potentially biasing estimates of NPC-specific survival.

In conclusion, we successfully estimated population-based NPC survival in endemic southern China using a passive-active-passive circle follow-up strategy that enabled highly complete follow-up in a truly representative cohort. We found that despite advances in radiotherapy techniques and economic development, which have most likely improved NPC survival throughout southern China, survival among patients in the general population is poorer than reported in publications describing medical-university-affiliated or province-level hospital-based patient cohorts. Moreover, we estimated that earlier diagnosis and improved access to medical resources in rural areas served by prefecture-level hospitals could lead to substantial reductions in NPC mortality.

By demonstrating a strategy to achieve highly complete population-based follow-up for cancer outcomes in southern China, our study provides a representative view of NPC survival in the past decade. Our finding that NPC survival is poorer in the general population than in hospital-based cohorts is as expected, although not previously established, and should be generalizable to other endemic regions or countries. These results also hint that the real-world benefits of newer therapies may be less than those seen in trials. Reduction of disparities in NPC mortality nationwide and worldwide will most likely require improved allocation of medical resources to facilitate more cancer screening and access to advanced radiotherapy facilities in remote areas.

## Supplementary information

Supplementary tables and figures

## Data Availability

The data without private data are available upon reasonable request to the corresponding authors.

## Code availability

The R code could be accessed on the GitHub website https://github.com/xinyueandtianliangle/NPC_overallSurvival/tree/main.
