# Supplementary material for: Population-based nasopharyngeal carcinoma survival in southern China
Source: Br J Cancer. 2025 Oct 29;134(1):99–107. doi: 10.1038/s41416-025-03232-w (PMC12764582; doi:10.1038/s41416-025-03232-w)
Supplement: Supplementary file 1 — Supplementary tables and figures [file 41416_2025_3232_MOESM1_ESM.docx]

**Real-world data of population-based nasopharyngeal carcinoma survival in southern China**

**Supplementary Appendix**

Table of Contents

[Methods S1. Systematic literature review of NPC survival in NPC high-risk areas 2](#_Toc151163691)

[Methods S2. Statistical methods to calculate avoidable deaths. 4](#_Toc151163692)

[Methods S3. Missing values 5](#_Toc151163693)

[Figure S1. Study population flowchart 6](#_Toc151163694)

[Figure S2. Timeline of study follow-up by area. 7](#_Toc151163695)

[Figure S3. Passive-active-passive circle follow-up strategy and distribution of follow-up methods by vital status at end of study. 8](#_Toc151163696)

[Figure S4. Distribution of follow-up for causes of death by study area. 10](#_Toc151163697)

[Figure S5. Kaplan-Meier estimates of NPC-specific survival treating 165 decedents with an unknown cause of death as deceased as non-NPC or NPC. 11](#_Toc151163698)

[Figure S6. Cumulative mortality curves for NPC-specific deaths and deaths from other causes. 13](#_Toc151163699)

[Table S1: Summary of systematic literature review of 5-year survival in NPC high-risk areas 15](#_Toc151163700)

[Table S2. Hazard ratios (HRs) in relation to demographic and clinical characteristics with NPC in southern China, from 2010-2013. 23](#_Toc151163701)

[Reference 27](#_Toc151163702)

# **Methods S1. Systematic literature review of NPC survival in NPC high-risk areas**

To compare our results with the previous literature, we searched MEDLINE and Web of Science for published estimates of NPC survival from 2009 to 19 August 2022 using the search strategy shown below. The results are summarized in Table S1.

Search string: (("mortality"[MeSH Subheading] OR "mortality"[All Fields] OR "survival"[All Fields] OR "survival"[MeSH Terms] OR "survivability"[All Fields] OR "survivable"[All Fields] OR "survivals"[All Fields] OR "survive"[All Fields] OR "survived"[All Fields] OR "survives"[All Fields] OR "surviving"[All Fields]) AND ("nasopharyngeal carcinoma"[MeSH Terms] OR ("nasopharyngeal"[All Fields] AND "carcinoma"[All Fields]) OR "nasopharyngeal carcinoma"[All Fields])) AND (clinicalstudy[Filter] OR clinicaltrial[Filter] OR clinicaltrialprotocol[Filter] OR clinicaltrialphasei[Filter] OR observationalstudy[Filter] OR randomizedcontrolledtrial[Filter])

# **Methods S2. Statistical methods to calculate avoidable deaths**

We first built a flexible parametric model including potential confounders, prognostic factors, and interaction terms. Covariates were further selected based on log-rank tests using the threshold of 0.05. Interaction terms were included according to plotting the log cumulative hazard, namely, log (-log(S(t))), on years since diagnosis. After checking the proportional hazards assumption and univariate p values, we built a flexible parametric model restricting our analysis to the first eight years after diagnosis, adjusting for age at diagnosis, sex, residential area, house type at diagnosis, attained education, occupation at diagnosis, smoking history, treatment hospital, BMI before treatment, KPS score, pathological classification, cancer stage, and treatment; and including interaction effects of residential area, treatment hospital, cancer stage, and treatment pattern. Only cases with non-missing values for the selected covariates were included. Second, we estimated predicted survival probabilities by setting the covariates to observed values, except that cancer stage was fixed to early stages (I/II); or treatment hospital was fixed to medical-university-affiliated/province-level. Finally, we averaged the predicted survival probabilities over the entire population and calculated the absolute difference over time by subtracting averaged survival probabilities between the fixed hypothetical scenario and the observed real-world scenario.

# **Methods S3. Missing values**

For covariates at baseline, there was 0.2% (6/2529) missing values of smoking history, 6.8% (173/2529) of treatment hospital, 9.3% (236/2529) of KPS before treatment, 6.8% (172/2529) of histological type, 7.0% (178/2529) of clinical stage, 7.6% (191/2529) treatment pattern and 6.6% (168/2529) of radiotherapy technique. We introduced one category missing for baseline covariates and calculated the survival probabilities within missing category (not shown in the results).

For 165 (6.5%) deceased cases with an unknown cause of death, we conducted multiple imputation, assuming that missing values in our data were missing at random (MAR). We checked the associations of missing patterns with covariates and cumulative hazards, it was related to residential areas, treatment hospital and occupation at diagnosis, indicating the missing pattern was covariates-dependent. The missing values of causes of deaths for those 165 cases were imputed from observed data using chained equations via *r* *mice* package. We used follow-up time, age at diagnosis, sex, marital status at diagnosis, education, occupation at diagnosis, smoking history, treatment hospital, BMI at diagnosis, KPS, pathological type, cancer stage, treatment pattern radiotherapy technique, nasopharyngeal radiotherapy dose, residential areas and cumulative hazards as predictors and logistic regression models for imputation, to generate five datasets. For these five imputed datasets, the Kaplan-Meier estimates, Cox proportional hazards regression models and flexible parametric models were performed and the results were pooled by Rubin’s formula. To check the robustness of the estimated results, we performed sensitivity analyses by single imputation considering that these 165 deceased cases died of NPC or non-NPC (**Figure S5**).

# **Methods S4. Power calculation**

The study population was the patient cohort of a population-based case-control study (NPCGEE). The number of sample size was pre-defined. Thus, we calculated the statistical power for the overall survival difference of cancer stage. The number of early-stage and late-stage were 289 and 2240 respectively. Given that the mortality of early-stage and late-stage were 0.2 and 0.8 at five years, the statistical power was 100%.

# **Figure S1. Study population flowchart**

# **Figure S2.** **Timeline of study follow-up by area**

Passive follow-up included linkages with regional cancer registries, the Total Population Registry, Death Registry, medical insurance system, and hospital medical records. Active follow-up included telephone calls to patients or their relatives, and home visits by village doctors.

# **Figure S3. Passive-active-passive circle follow-up strategy and distribution of follow-up methods by vital status at end of study**

Panel A shows the passive-active-passive circle follow-up strategy. Panel B shows the distribution of follow-up methods stratified by vital status at the end of the study: alive (left) or dead (right).

Passive follow-up included linkages with regional cancer registries, the Total Population Registry, Death Registry, medical insurance system, and hospital medical records. Active follow-up included telephone calls to patients or their relatives, and home visits by village doctors.

# **Figure S4. Distribution of follow-up for causes of death by study area**


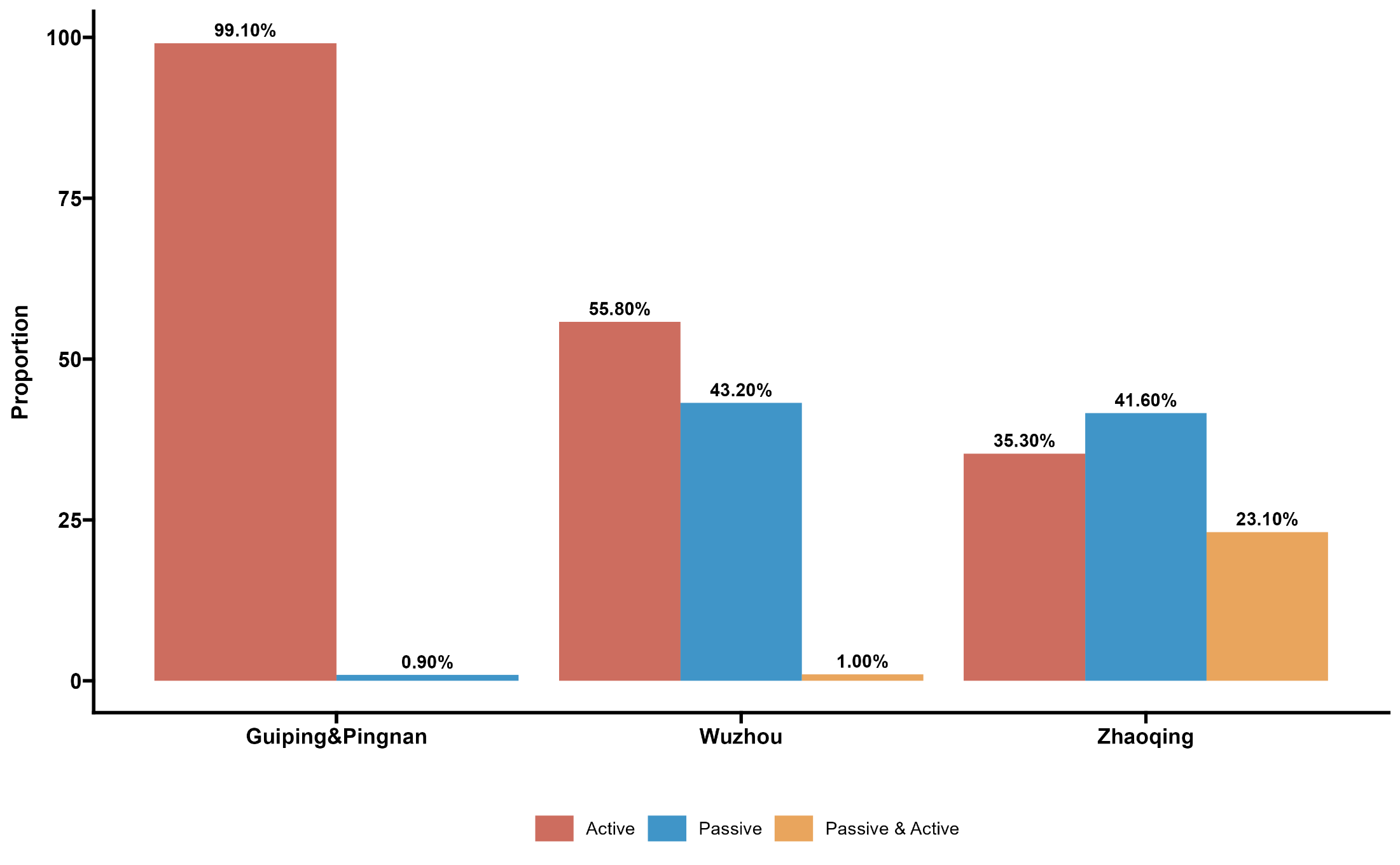


# **Figure S5. Kaplan-Meier estimates of NPC-specific survival treating 165 decedents with an unknown cause of death as deceased as non-NPC or NPC**

Kaplan-Meier estimates of NPC-specific survival curves when all the 165 deceased cases with unknown cause were regarded as dying from non-NPC (A1-A4) and from NPC (B1-B4).

Abbreviations: NPC, nasopharyngeal carcinoma; DDS, disease/NPC-specific survival.


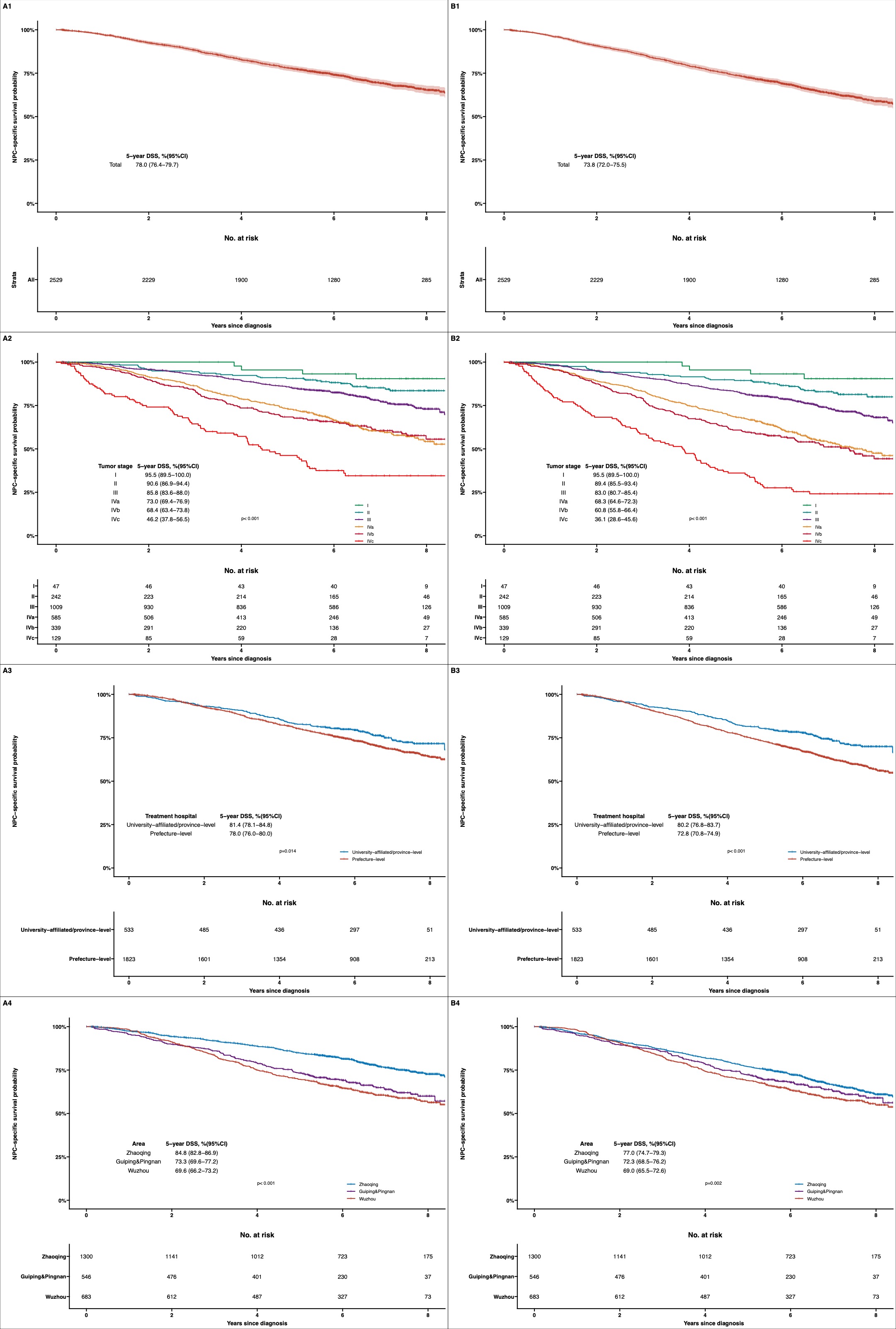


# **Figure S6. Cumulative mortality curves for NPC-specific deaths and deaths from other causes**

Panel A: 165 decedents with an unknown cause of death were treated as dying from other causes. Panel B: 165 decedents with an unknown cause of death were treated as dying from NPC.

A


B

# **Table S1: Summary of systematic literature review of 5-year survival in NPC high-risk areas**

| **First author** | **Design** | **Study base** | **Follow up duration (median, range)** | **Number of study population** | **Diagnosis year** | **Data source** | **Study area** | **Diagnosis age (median: range)** | **Male** | **Female** | **TNM stage version** | **Stage distribution (%)** | **Five-year survival (%)** |
| --- | --- | --- | --- | --- | --- | --- | --- | --- | --- | --- | --- | --- | --- |
| This research | Prospective  cohort study | Population-based | 72 (0-106) | 2529 | 2010-2013 | Guangdong & Guangxi | Guangdong & Guangxi | 48 (20-74) | 1856 | 673 | 7th AJCC | I: 1.8 II: 9.5 III: 39.9 IVa: 23.1 IVb: 13.4 Metastasis: 5.1 Missing: 7.2 | Stage I: 91.1 (83.2-99.8) Stage II: 88.1 (84.1-92.4) Stage III: 79.8 (77.4-82.4) Stage IVa:63.8 (60.0-67.9) Stage IVb: 57.7 (52.7-63.3) Metastasis: 34.4 (27.1-43.7)  Overall: 70.1 (68.4-72.0) |
| Sun et al. ^1^ | Retrospective cohort study | Hospital-based | 62 (1-296) | 5626 | 2008-2012 | SYSUCC | Guangdong |  | 4143 | 1483 | 7th AJCC | I: 3.2 II: 13.8 III: 54.0 IVa: 20.8 IVb: 8.3 Metastasis: 0 | Stage I: 100 Stage II: 94.6 (92.2-97.0) Stage III: 88.1 (86.5-89.7) Stage IVa: 80.5 (77.6,83.4) Stage IVb: 72.1 (66.6-77.6) |
| Sun et al. ^1^ | Retrospective cohort study | Hospital-based | 62 (1-296) | 4755 | 2003-2007 | SYSUCC | Guangdong |  | 3564 | 1191 | 7th AJCC | I: 3.7 II: 17.5 III: 49.5 IVa: 22.6 IVb: 6.7 Metastasis: 0 | Stage I: 97.7 (95.5-99.9) Stage II: 91.7 (89.7-93.7) Stage III: 81.2 (79.6-82.8) Stage IVa: 72.1 (69.4-74.8) Stage IVb: 57.1 (51.6- 62.6) |
| Sun et al. ^1^ | Retrospective cohort study | Hospital-based | 62 (1-296) | 3884 | 1997-2002 | SYSUCC | Guangdong |  | 2977 | 907 | 7th AJCC | I: 2.0 II: 25.6 III: 44.6 IVa: 17.6 IVb: 7.3 Metastasis: 0 | Stage I: 91.6 (87.7-95.5) Stage II: 80.8 (78.3-83.3) Stage III: 65.8 (63.4-68.2) Stage IVa: 51.2 (47.3-55.1) Stage IVb: 42.1 (36.2-48.0) |
| Sun et al. ^1^ | Retrospective cohort study | Hospital-based | 62 (1-296) | 6000 | 1990-1996 | SYSUCC | Guangdong |  | 4559 | 1441 | 7th AJCC | I: 3.0 II: 20.2 III: 51.4 IVa: 17.3 IVb: 8.0 Metastasis: 0 | Stage I: 86.2 (81.1-91.3) Stage II: 78.7 (76.3-81.1) Stage III: 65.2 (63.4-67.0) Stage IVa: 48.9 (45.8-52.0) Stage IVb: 38.7(34.0-43.4) |
| Peng et al. ^2^ | Retrospective cohort study | Hospital-based | 42 (1-65) | 296 | 2012-2015 | SYSUCC | Guangdong |  | 236 | 60 | 7th AJCC | I: 0 II: 0 III: 48.3 IV: 51.7  Metastasis: 0 | Three-year overall survival: 93.1 |
| Sun et al. ^3^ | Retrospective cohort study | Hospital-based | 27 (1-138) | 266 | 2006-2016 | SYSUCC | Guangdong | 47 | 218 | 48 |  | Metastasis: 100 | Three-year survival: 52.6/50.3 |
| Tao et al. ^4^ | propensity scorematching analysis | Hospital-based | 60 (5-124) | 801 | 2009-2016 | Affiliated Cancer Hospital & Institute of Guangzhou Medical University | Guangdong |  | 590 | 211 | 8th AJCC | I: 0 II: 0 III: 64.7 IV: 35.3 Metastasis: 0 | Overall: 84.2 |
| Du et al.^5^ | Retrospective cohort study | Hospital-based | 65 (6-83) | 149 | 2010-2011 | Zhongshan City People’s Hospital | Guangdong | 45 (12-74) | 119 | 30 | 7th AJCC | I: 6 II: 14.1 III: 36.2 IV: 43.6 Metastasis: 0 | Overall: 80.5 Stage I: 100.0 Stage II: 95.2 Stage III: 87.0 Stage IV: 67.2 |
| Qin et al. ^6^ | Retrospective cohort study | Hospital-based | 54 (11-85) | 249 | 2006-2008 | First Affiliated Hospital of Guangxi Medical University | Guangxi |  | 198 | 51 | 6th AJCC | I: 0 II: 0 III: 56.6 IV: 43.4 Metastasis: 0 | Overall: 78.4 |
| Zhao et al. ^7^ | Retrospective cohort study | Hospital-based | 38 (4-97) | 527 | 2007-2011 | Cancer Hospital of Guangxi Medical University | Guangxi | 44 (16-79) | 394 | 133 | 7th AJCC | I: 3.4 II: 21.1 III: 46.5 IV: 29.0 Metastasis: 0 | Overall: 80.9 Stage I: 100 Stage II: 87.4 Stage III: 84.3 Stage IV: 67.1 |
| Chan et al. ^8^ | propensity scorematching analysis | Population-based |  | 237 | 2006-2017 | Cancer Registry | Hongkong | 51 (16-85) | 182 | 55 | 8th AJCC | I: 1.3 II: 10.1 III: 46.4 IVa: 35.0 IVb/Metastasis: 7.2 | Overall: 79.3 (73.4- 85.2) |
| Lee et al. ^9^ | Retrospective cohort study | Hospital-based |  | 2867 | 1996-2000 | Five hospitals | Hongkong | 47 | 2064 | 803 | 5th AJCC | I: 7.0 II: 41.0 III: 25.0 IV: 28.0 Metastasis: 0 | Overall: 75.0 Stage I: 90.0 Stage II: 84.0 Stage III: 75.0 Stage IV: 58.0 |
| Zong et al. ^10^ | Retrospective cohort study | Hospital-based | 57 (2-102) | 1241 | 2005-2010 | Fujian Provincial Cancer Hospital | Fujian | 46 (11-84) | 938 | 303 | 7th AJCC | I: 4.8 II: 26.2 III: 45.4 IVa: 18.4 IVb: 5.2 Metastasis: 0 | Overall: 81.1 Stage I: 98.3 Stage II: 89.3 Stage III: 80.8 Stage IVa: 68.7 Stage IVb: 68.5 |
| Lin et al. ^11^ | Retrospective cohort study | Hospital-based | 22 (2-142) | 105 | 1995-2002 | Hospitals | Fujian | 46 | 89 | 16 | 5th AJCC | Metastasis: 100 | Overall: 16.6 |
| Pan et al. ^12^ | Retrospective cohort study | Hospital-based |  | 62 | 2007-2008 | Southwest Hospital | Chongqing | 47 (23-71) | 47 | 15 | 6th AJCC | I: 0 II: 0 III: 59.7 IVa: 40.3 Metastasis: 0 | Overall: 75.8 |
| Huang et al. ^13^ | Retrospective cohort study | Hospital-based | 51 (10-106) | 608 | 2008-2013 | Tongji Medical College, Huazhong University of Science and Technology | Hubei | 48 (11-79) | 426 | 182 | 7th AJCC | I: 1.3 II: 8.4 III: 53.0 IVa: 22.9 IVb: 14.5 Metastasis: 0 | Overall: 79.7 Stage I: 100 Stage II: 92.2 Stage III: 88.4 Stage IVa: 74.3 Stage IVb: 48.6 |
| Feng et al. ^14^ | Retrospective cohort study | Hospital-based | 63 (9-82) | 363 | 2003-2005 | Sichuan Cancer Hos- pital | Sichuan | 48 (16-78) | 285 | 78 | 6th AJCC | I: 4.4 II: 25.9 III: 45.4 IV: 24.3 Metastasis: 0 | Overall: 79.3 |
| Dwijayanti et al. ^15^ | Retrospective cohort study | Hospital-based | 44 (1-60) | 261 | 2009-2013 | Dharmais Cancer Hospital | Indonesia |  |  |  | 7th AJCC | I: 0 II: 0 III: 34.9 IV: 65.1 Metastasis: 0 | Overall: 38.6 |
| Abdullah et al. ^16^ | Retrospective cohort study | Hospital-based |  | 266 | 2008-2012 | ubang Jaya Medical Centre | Malaysia |  | 183 | 83 | NA | I: 8.0 II: 23.0 III: 44.0 IVa: 8.0 IVb: 12.0 IVc: 4.0 Metastasis: 4.0 | Overall: 73 Stage I: 100 Stage II: 91 Stage III: 72 Stage IV: 44 |
| Mak et al. ^17^ | Retrospective cohort study | Hospital-based |  | 558 | 2002-2012 | National University Hospital | Singapore | 52 | 409 | 149 | 7th AJCC | I: 9.3 II: 24.6 III: 30.6 IVa: 16.1 IVb: 9.9 IVc: 9.5 Metastasis: 9.5 | Overall: 69.9 |

# **Table S2. Hazard ratios (HRs) in relation to demographic and clinical characteristics with NPC in southern China, from 2010-2013**

|  | | | **All-cause mortality** | | **NPC-specific mortality^**^** | |
| --- | --- | --- | --- | --- | --- | --- |
|  | **Death** | **Person years** | **Crude HR   (95%CI)** | **Adjusted HR   (95%CI)^*^** | **Crude HR   (95%CI)** | **Adjusted HR   (95%CI)^*^** |
| Age at cancer diagnosis | 1,019 | 13,907 | 1.04 (1.03-1.04) | 1.03 (1.02-1.04) | 1.03 (1.03-1.04) | 1.03 (1.02-1.03) |
| Residential areas |  |  |  |  |  |  |
| Zhaoqing | 481 | 7,374 | Ref | Ref | Ref | Ref |
| Guiping&Pingnan | 228 | 2,833 | 1.26 (1.07-1.47) | 1.36 (1.13-1.65) | 1.26 (1.05-1.50) | 1.38 (1.11-1.71) |
| Wuzhou | 310 | 3,700 | 1.30 (1.13-1.50) | 1.20 (1.04-1.39) | 1.41 (1.21-1.66) | 1.30 (1.10-1.53) |
| Sex |  |  |  |  |  |  |
| Female | 205 | 3,942 | Ref | Ref | Ref | Ref |
| Male | 814 | 9,965 | 1.59 (1.36-1.85) | 1.39 (1.13-1.72) | 1.46 (1.24-1.73) | 1.28 (1.01-1.63) |
| Marital status at diagnosis |  |  |  |  |  |  |
| Married | 958 | 13,102 | Ref | Ref | Ref | Ref |
| Not married | 61 | 804 | 1.03 (0.80-1.34) | 1.18 (0.90-1.55) | 0.95 (0.70-1.28) | 1.06 (0.78-1.45) |
| Educational attainment |  |  |  |  |  |  |
| Illiterate/Primary school | 449 | 5,340 | Ref | Ref | Ref | Ref |
| Middle school | 400 | 5,633 | 0.84 (0.73-0.96) | 0.92 (0.80-1.06) | 0.84 (0.72-0.98) | 0.91 (0.78-1.07) |
| High school | 148 | 2,249 | 0.78 (0.64-0.93) | 0.86 (0.70-1.04) | 0.74 (0.60-0.91) | 0.79 (0.63-0.99) |
| Vocational or technical college/University and above | 22 | 683 | 0.37 (0.24-0.57) | 0.63 (0.40-1.00) | 0.34 (0.21-0.56) | 0.55 (0.33-0.93) |
| Occupation at diagnosis |  |  |  |  |  |  |
| Farmer | 367 | 4,633 | Ref | Ref | Ref | Ref |
| Blue collar | 393 | 5,687 | 0.87 (0.76-1.00) | 1.13 (0.97-1.33) | 0.88 (0.75-1.03) | 1.15 (0.96-1.37) |
| White collar | 111 | 2,001 | 0.69 (0.56-0.86) | 1.01 (0.80-1.28) | 0.73 (0.57-0.92) | 1.11 (0.86-1.43) |
| Unemployed | 24 | 456 | 0.65 (0.43-0.99) | 0.95 (0.63-1.45) | 0.66 (0.41-1.04) | 0.99 (0.62-1.57) |
| Unknown/other | 124 | 1,129 | 1.40 (1.14-1.72) | 1.15 (0.93-1.42) | 1.38 (1.09-1.75) | 1.16 (0.90-1.48) |
| Smoking history |  |  |  |  |  |  |
| Never | 370 | 6,455 | Ref | Ref | Ref | Ref |
| Former | 82 | 999 | 1.43 (1.13-1.82) | 1.04 (0.80-1.36) | 1.28 (0.97-1.70) | 1.00 (0.74-1.36) |
| Current | 563 | 6,425 | 1.55 (1.36-1.77) | 1.06 (0.90-1.26) | 1.51 (1.30-1.74) | 1.09 (0.89-1.33) |
| Missing | 4 | 26 | 2.79 (1.04-7.47) | 2.26 (0.82-6.18) | 2.78 (0.89-8.66) | 2.55 (0.80-8.13) |
| Treatment hospitals |  |  |  |  |  |  |
| Medical university-affiliated/province-level | 160 | 3,056 | Ref | Ref | Ref | Ref |
| Prefecture-level | 768 | 9,977 | 1.48 (1.25-1.75) | 1.42 (1.16-1.73) | 1.47 (1.22-1.77) | 1.38 (1.10-1.72) |
| Missing | 91 | 874 | 2.02 (1.56-2.62) | 2.04 (1.06-3.92) | 1.73 (1.28-2.33) | 1.82 (0.86-3.84) |
| BMI before treatment |  |  |  |  |  |  |
| Normal weight | 498 | 6,520 | Ref | Ref | Ref | Ref |
| Underweight | 153 | 1,633 | 1.23 (1.03-1.48) | 1.03 (0.86-1.25) | 1.26 (1.03-1.54) | 1.04 (0.84-1.28) |
| Overweight | 212 | 3,835 | 0.72 (0.61-0.84) | 0.73 (0.62-0.87) | 0.69 (0.58-0.83) | 0.72 (0.60-0.86) |
| Obese | 37 | 734 | 0.65 (0.46-0.91) | 0.78 (0.56-1.10) | 0.63 (0.43-0.91) | 0.77 (0.53-1.13) |
| Missing | 119 | 1,183 | 1.33 (1.09-1.62) | 0.92 (0.62-1.39) | 1.22 (0.97-1.54) | 1.08 (0.70-1.66) |
| KPS before treatment |  |  |  |  |  |  |
| < 90 | 157 | 1,560 | Ref | Ref | Ref | Ref |
| ≥ 90 | 749 | 11,102 | 0.66 (0.55-0.78) | 0.84 (0.70-1.01) | 0.62 (0.51-0.75) | 0.78 (0.64-0.96) |
| Missing | 113 | 1,244 | 0.89 (0.70-1.14) | 0.79 (0.52-1.20) | 0.72 (0.54-0.96) | 0.60 (0.37-0.98) |
| Histological type |  |  |  |  |  |  |
| Others | 48 | 495 | Ref | Ref | Ref | Ref |
| Non-keratinizing carcinoma | 885 | 12,501 | 0.73 (0.55-0.98) | 0.82 (0.61-1.11) | 0.79 (0.56-1.11) | 0.89 (0.63-1.26) |
| Missing | 86 | 910 | 0.98 (0.69-1.40) | 0.57 (0.33-0.96) | 0.90 (0.60-1.36) | 0.53 (0.29-0.98) |
| Cancer stage |  |  |  |  |  |  |
| I | 6 | 320 | Ref | Ref | Ref | Ref |
| II | 47 | 1,537 | 1.65 (0.71-3.87) | 1.98 (0.85-4.65) | 1.96 (0.70-5.50) | 2.38 (0.85-6.70) |
| III | 303 | 5,939 | 2.81 (1.25-6.30) | 3.32 (1.48-7.47) | 3.34 (1.24-8.97) | 3.90 (1.45-10.50) |
| IVa | 292 | 3,022 | 5.47 (2.44-12.28) | 5.46 (2.42-12.29) | 6.63 (2.47-17.83) | 6.47 (2.40-17.44) |
| IVb | 181 | 1,704 | 6.04 (2.68-13.62) | 6.52 (2.88-14.76) | 7.51 (2.78-20.29) | 7.99 (2.94-21.66) |
| IVc | 97 | 478 | 12.16 (5.33-27.76) | 11.87 (5.18-27.21) | 16.70 (6.12-45.53) | 16.26 (5.93-44.56) |
| Missing | 93 | 903 | 5.79 (2.54-13.23) | 5.77 (2.11-15.78) | 6.28 (2.29-17.22) | 7.99 (2.43-26.26) |
| Treatment pattern |  |  |  |  |  |  |
| CCRT | 384 | 6,027 | Ref | Ref | Ref | Ref |
| CCRT+ICT/ACT | 354 | 5,270 | 1.07 (0.93-1.24) | 0.98 (0.85-1.14) | 1.05 (0.89-1.23) | 0.96 (0.81-1.13) |
| RT only | 90 | 1,153 | 1.23 (0.98-1.54) | 1.24 (0.98-1.58) | 1.20 (0.92-1.57) | 1.22 (0.92-1.62) |
| Chemo only | 59 | 258 | 3.99 (3.03-5.25) | 2.18 (1.61-2.94) | 4.30 (3.21-5.78) | 2.25 (1.63-3.10) |
| Neither RT nor CT | 32 | 228 | 2.29 (1.60-3.29) | 1.34 (0.91-1.96) | 2.56 (1.75-3.74) | 1.42 (0.95-2.12) |
| RT+ICT/ACT | 0 | 6 | 0.00 (0.00-Inf) | 0.00 (0.00-Inf) | 0.00 (0.00-Inf) | 0.00 (0.00-Inf) |
| Missing | 100 | 961 | 1.67 (1.34-2.08) | 1.58 (0.85-2.93) | 1.45 (1.12-1.88) | 1.69 (0.83-3.45) |
| RT technique |  |  |  |  |  |  |
| 2DRT | 578 | 7,338 | Ref | Ref | Ref | Ref |
| 3DRT | 39 | 748 | 0.67 (0.49-0.93) | 0.77 (0.55-1.07) | 0.61 (0.42-0.89) | 0.70 (0.48-1.03) |
| IMRT | 212 | 4,361 | 0.62 (0.53-0.73) | 0.69 (0.58-0.82) | 0.63 (0.53-0.75) | 0.70 (0.58-0.84) |
| No RT | 91 | 486 | 2.54 (2.04-3.18) | 1.59 (1.24-2.03) | 2.81 (2.22-3.56) | 1.68 (1.29-2.18) |
| Unknown technique | 7 | 146 | 0.60 (0.29-1.27) | 0.65 (0.29-1.46) | 0.59 (0.25-1.40) | 0.65 (0.25-1.66) |
| Missing | 92 | 825 | 1.45 (1.16-1.81) | 1.57 (0.68-3.67) | 1.27 (0.98-1.64) | 1.52 (0.59-3.95) |
| Nasopharyngeal radiation dose |  |  |  |  |  |  |
| < 70 Gy | 148 | 2,369 | Ref | Ref | Ref | Ref |
| ≥ 70 Gy | 665 | 9,924 | 1.05 (0.88-1.26) | 1.10 (0.91-1.32) | 0.96 (0.79-1.16) | 1.00 (0.82-1.23) |
| No radiotherapy | 91 | 486 | 3.16 (2.43-4.10) | 1.86 (1.41-2.47) | 3.24 (2.45-4.29) | 1.86 (1.38-2.51) |
| Missing | 115 | 1,126 | 1.64 (1.28-2.09) | 1.06 (0.67-1.67) | 1.39 (1.06-1.83) | 1.08 (0.66-1.78) |
| ^*^HRs was adjusted for age at diagnosis, sex, residential area, educational attainment, marital status at diagnosis, smoking history, alcohol consumption, calendar year at diagnosis, BMI before treatment, KPS before treatment and cancer stage. | | | | | | |
|  |  |  |  |  |  |  |
| ^**^Combined results from multiple imputation for 165 deceased cases with unknown cause | | | | | | |
| Abbreviations: NPC, nasopharyngeal carcinoma; CI, confidence interval; BMI, body mass index; KPS, Karnofsky performance scale. | | | | | | |

# **Reference**

1. Sun XSS, Liu SLL, Luo MJJ, et al. The Association Between the Development of Radiation Therapy, Image Technology, and Chemotherapy, and the Survival of Patients With Nasopharyngeal Carcinoma: A Cohort Study From 1990 to 2012. *Int J Radiat Oncol Biol Phys*. 2019;105(3):581-590. doi:10.1016/j.ijrobp.2019.06.2549

2. Peng H, Tang LLL, Liu X, et al. Anti-EGFR targeted therapy delivered before versus during radiotherapy in locoregionally advanced nasopharyngeal carcinoma: a big-data, intelligence platform-based analysis. *BMC Cancer*. 2018;18(1). doi:10.1186/s12885-018-4268-y

3. Sun XSS, Wang XHH, Liu SLL, et al. Comparison of Gemcitabine Plus Cisplatin vs. Docetaxel Plus Fluorouracil Plus Cisplatin Palliative Chemotherapy for Metastatic Nasopharyngeal Carcinoma. *Front Oncol*. 2020;10. doi:10.3389/fonc.2020.01295

4. Tao HYY, Zhan ZJJ, Qiu WZZ, et al. Clinical value of docetaxel plus cisplatin (TP) induction chemotherapy followed by TP concurrent chemoradiotherapy in locoregionally advanced nasopharyngeal carcinoma. *J Cancer*. 2021;12(1):18-27. doi:10.7150/jca.49944

5. Du Y, Zhang W, Lei F, et al. Long-term survival after nasopharyngeal carcinoma treatment in a local prefecture-level hospital in southern China. *Cancer Manag Res*. 2020;12:1329-1338. doi:10.2147/CMAR.S237278

6. Qin L, Wu F, Lu H, Wei B, Li G, Wang R. Tumor Volume Predicts Survival Rate of Advanced Nasopharyngeal Carcinoma Treated with Concurrent Chemoradiotherapy. *Otolaryngology - Head and Neck Surgery (United States)*. 2016;155(4):598-605. doi:10.1177/0194599816644408

7. Zhao W, Lei H, Zhu X, Li L, Qu S, Liang X. Investigation of long-term survival outcomes and failure patterns of patients with nasopharyngeal carcinoma receiving intensity-modulated radiotherapy: a retrospective analysis. *Oncotarget*. 2016;7(52):86914-86925. doi:10.18632/ONCOTARGET.13564

8. Chan SK, Chau SC, Chan SY, et al. Incidence and Demographics of Nasopharyngeal Carcinoma in Cheung Chau Island of Hong Kong-A Distinct Geographical Area With Minimal Residential Mobility and Restricted Public Healthcare Referral Network. *Cancer Control*. 2021;28. doi:10.1177/10732748211047117

9. Lee AWM, Sze WM, Au JSK, et al. Treatment results for nasopharyngeal carcinoma in the modern era: the Hong Kong experience. *Int J Radiat Oncol Biol Phys*. 2005;61(4):1107-1116. doi:10.1016/J.IJROBP.2004.07.702

10. Zong J, Lin S, Lin J, et al. Impact of intensity-modulated radiotherapy on nasopharyngeal carcinoma: Validation of the 7th edition AJCC staging system. *Oral Oncol*. 2015;51(3):254-259. doi:10.1016/J.ORALONCOLOGY.2014.10.012

11. Lin S, Tham IWK, Pan J, Han L, Chen Q, Lu JJ. Combined high-dose radiation therapy and systemic chemotherapy improves survival in patients with newly diagnosed metastatic nasopharyngeal cancer. *American Journal of Clinical Oncology: Cancer Clinical Trials*. 2012;35(5):474-479. doi:10.1097/COC.0B013E31821A9452

12. Pan F, Ruan Z, Li J, et al. Radiotherapy combined docetaxel and oxaliplatin chemotherapy is effective in patients with locally advanced nasopharyngeal carcinoma. *Medical Oncology*. 2015;32(11). doi:10.1007/S12032-015-0698-4

13. Huang J, Yang Z yong, Wu B, et al. Long-term Therapeutic Outcome and Prognostic Factors of Patients with Nasopharyngeal Carcinoma Receiving Intensity-modulated Radiotherapy: An Analysis of 608 Patients from Low-endemic Regions of China. *Curr Med Sci*. 2021;41(4):737-745. doi:10.1007/S11596-021-2405-3

14. Feng M, Wang W, Fan Z, et al. Tumor volume is an independent prognostic indicator of local control in nasopharyngeal carcinoma patients treated with intensity-modulated radiotherapy. *RADIATION ONCOLOGY*. 2013;8(1). doi:10.1186/1748-717X-8-208

15. Dwijayanti F, Prabawa A, Besral, Herawati C. The Five-Year Survival Rate of Patients with Nasopharyngeal Carcinoma Based on Tumor Response after Receiving Neoadjuvant Chemotherapy, Followed by Chemoradiation, in Indonesia: A Retrospective Study. *Oncology*. 2020;98(3):154-160. doi:10.1159/000504449

16. Abdullah MM, Foo YC, Yap BK, Lee CML, Hoo LP, Lim TO. Retrospective Analysis of Cancer Care Performance and Survival Outcome for Nasopharyngeal Carcinoma at a leading Cancer Treatment Centre in Malaysia 2008-2012. *Asian Pac J Cancer Prev*. 2019;20(6):1701. doi:10.31557/APJCP.2019.20.6.1701

17. Mak HW, Lee SH, Chee J, et al. Clinical Outcome among Nasopharyngeal Cancer Patients in a Multi-Ethnic Society in Singapore. *PLoS One*. 2015;10(5). doi:10.1371/JOURNAL.PONE.0126108
